# Chemical genetic-based phenotypic screen reveals novel regulators of gluconeogenesis in human primary hepatocytes

**DOI:** 10.1038/s41525-018-0062-7

**Published:** 2018-08-15

**Authors:** Haixia Zou, Qian Liu, Li Meng, Jingye Zhou, Chenxiao Da, Xikun Wu, Lichun Jiang, Jianyong Shou, Haiqing Hua

**Affiliations:** Lilly China Research and Development Center (LCRDC), Eli Lilly & Company, Shanghai, China

## Abstract

Insulin resistance is a pathophysiological hallmark of type 2 diabetes and nonalcoholic fatty liver disease. Under the condition of fat accumulation in the liver, suppression of hepatic glucose production by insulin is diminished. In order to gain deeper understanding of dysregulation of glucose production in metabolic diseases, in the present study, we performed an unbiased phenotypic screening in primary human hepatocytes to discover novel mechanisms that regulate gluconeogenesis in the presence of insulin. To optimize phenotypic screening process, we used a chemical genetic screening approach by building a small-molecule library with prior knowledge of activity-based protein profiling. The “positive hits” result from the screen will be small molecules with known protein targets. This makes downstream deconvolution process (i.e., determining the relevant biological targets) less time-consuming. To unbiasedly decipher the molecular targets, we developed a novel statistical method and discovered a set of genes, including DDR3 and CACNA1E that suppressed gluconeogenesis in human hepatocytes. Further investigation, including transcriptional profiling and gene network analysis, was performed to understand the molecular functions of DRD3 and CACNA1E in human hepatocytes.

## Diabetes: Chemical genetic screen reveals potential drug targets

A high-throughput screening protocol helped identify two novel genes involved in sugar metabolism that could be druggable targets for treating type 2 diabetes. Haiqing Hua and colleagues from the Lilly China Research and Development Center, a division of Eli Lilly & Company in Shanghai, evaluated the ability of human liver cells to produce glucose when exposed to a library of more than 1500 small-molecule drugs with known molecular targets. The researchers distinguished a set of genes that, when inhibited, dramatically suppressed glucose production. Two of these genes had not previously been implicated in liver function. The researchers began to dissect that molecular function through studies of gene expression and gene network analysis. The results demonstrate a way to combine chemical genetics and phenotypic screening to identify novel drug targets, revealing two for diabetes.

## Introduction

Type 2 diabetes mellitus (T2DM) is a multifactorial disease, represented by heterogeneous patient populations with various degrees of obesity, insulin resistance, and beta cell dysfunction.^1^ Among these factors, insulin resistance is an independent risk factor of progressive deterioration from glucose intolerance to diabetes.^2^ Liver steatosis induces hepatic insulin resistance and strongly correlates with prediabetes and T2DM.^3^ Hepatic insulin resistance leads to uncontrolled hepatic gluconeogenesis. Therefore, therapeutics that improve hepatic insulin sensitivity and/or suppress gluconeogenesis may be beneficial for the treatment of T2DM. Phenotypic screen has been demonstrated to be a powerful approach to discover novel drug targets and molecules. For instance, FGF-21 was discovered to be a potent regulator of glucose uptake phenotype in mouse 3T3-L1.^4^ One challenge of small-molecule library-based phenotypic screening is the difficulty to decipher the targets of effective small molecules, though modern technologies including mRNA, protein, or image-based profiling greatly facilitate and accelerate the deconvolution process.^5^ On the other hand, functional genetics approaches (e.g., siRNA and CRISPR) are used to identify potential drug targets,^6^ but large amount of effort and time-consuming processes are required to select the appropriate targets and develop specific molecules for those targets.

In recent years, with the advance of human genome sequencing technology and accumulated knowledge of small-molecule libraries, chemical genetics has emerged as a powerful approach to discover potential drug targets.^7^ Traditional genetics approach uses gene knockout or RNAi technology to manipulate gene function and screens for correlations between gene function and a particular phenotype. Chemical genetics uses small molecules to manipulate protein function and screen for correlations between protein function and a particular phenotype. In the present study, we used chemical genetics approach to seek for protein–phenotype correlations that could relate insulin’s action on human hepatocytes with a set of biological targets (regulators of gluconeogenesis). Then, we used “specific modulators” (small molecules that well validated to specifically inhibit or activate our target proteins of interest) of the targets to further confirm the function of identified biological targets. Among the confirmed target genes, the functions of DRD3 and CACNA1E in hepatocytes have not been extensively elucidated before. Therefore, we performed a gene array study to further understand the mechanisms by which they manipulate glucose homeostasis in human hepatocytes. In the gene array study, we have chosen 84 genes whose function is related to insulin signaling and gluconeogenesis. We used quantitative PCR to detect the mRNA levels of these 84 genes before and after treatment of the “specific modulators”. Our data demonstrated that both DRD3 and CACNA1E regulate multiple genes in the pathway of glucose metabolism. For instance, glucokinase, IRS4, and/or IRS2 are upregulated more than twofold when the activity of DRD3 or CACNA1E are inhibited. Therefore, DRD3 and CACNA1E are potential therapeutic targets for the treatment of T2DM.

## Results

### Establish a chemical genetic screening approach in human primary hepatocytes

**Fig. 1:**
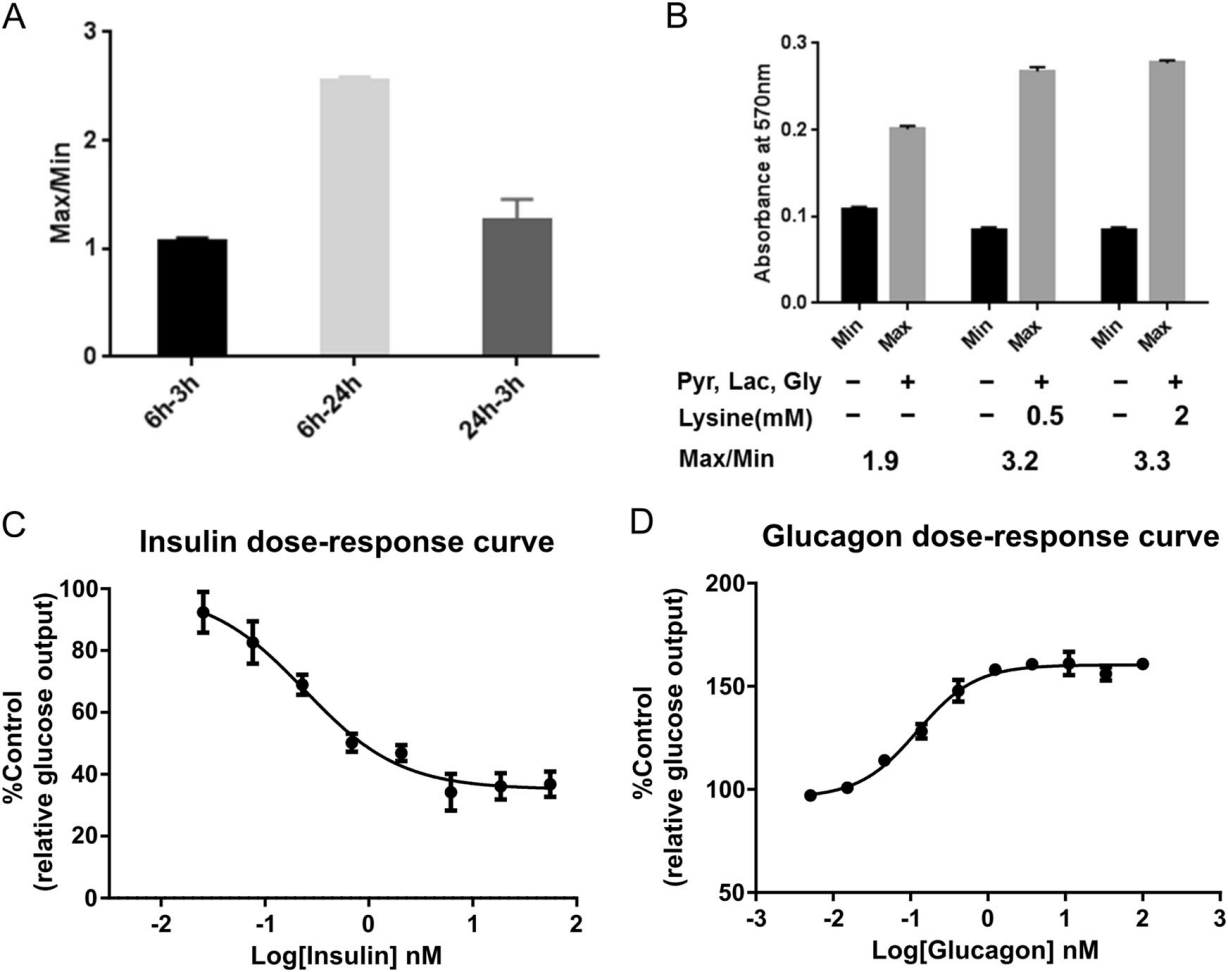
Optimize glucose production assay for primary human hepatocytes. **a** Ratio of MAX and MIN raw signal at indicated time points. Min: basal glucose signal without gluconeogenic substrates treatment; Max: glucose signal with gluconeogenic substrates treatment. 6–3 h/6–24 h: starve cells for 6 h and treat cells with gluconeogenic substrates for 3 or 24 h, respectively; 24–3h: starve cells for 24 h and treat cells with gluconeogenic substrates for 3 h. (*n* = 3). **b** Absorbance at 570 nm when treating human primary hepatocytes with pyruvate, lactate, glycine with or without lysine. (*n* = 3). **c** Dose response of gluconeogenesis inhibition by insulin. (*n* = 3). **d** Dose response of gluconeogenesis stimulation by glucagon. (*n* = 3)

In order to identify genes that are involved in gluconeogenesis in the presence of insulin, we first optimized glucose production assay for primary human hepatocytes. We tested glucose production at different time points following starvation and gluconeogenic substrates treatment to identify a study condition with the best signal-to-noise ratio (Fig. 1a). We found that 6-h starvation and 24-h substrates treatment was the optimal time window for the detection of glucose production from human primary hepatocytes. In addition to the commonly used gluconeogenesis substrates (pyruvate, lactate, and glycerol), we found that addition of lysine significantly increased signal window (Fig. 1b). With the optimized assay format, we screened hepatocytes from various human donors for their sensitivity to insulin and glucose. A batch of hepatocytes from a single donor that showed response to physiological levels of insulin (Fig. 1c) and glucagon (Fig. 1d) was identified and used for this study.

**Fig. 2:**
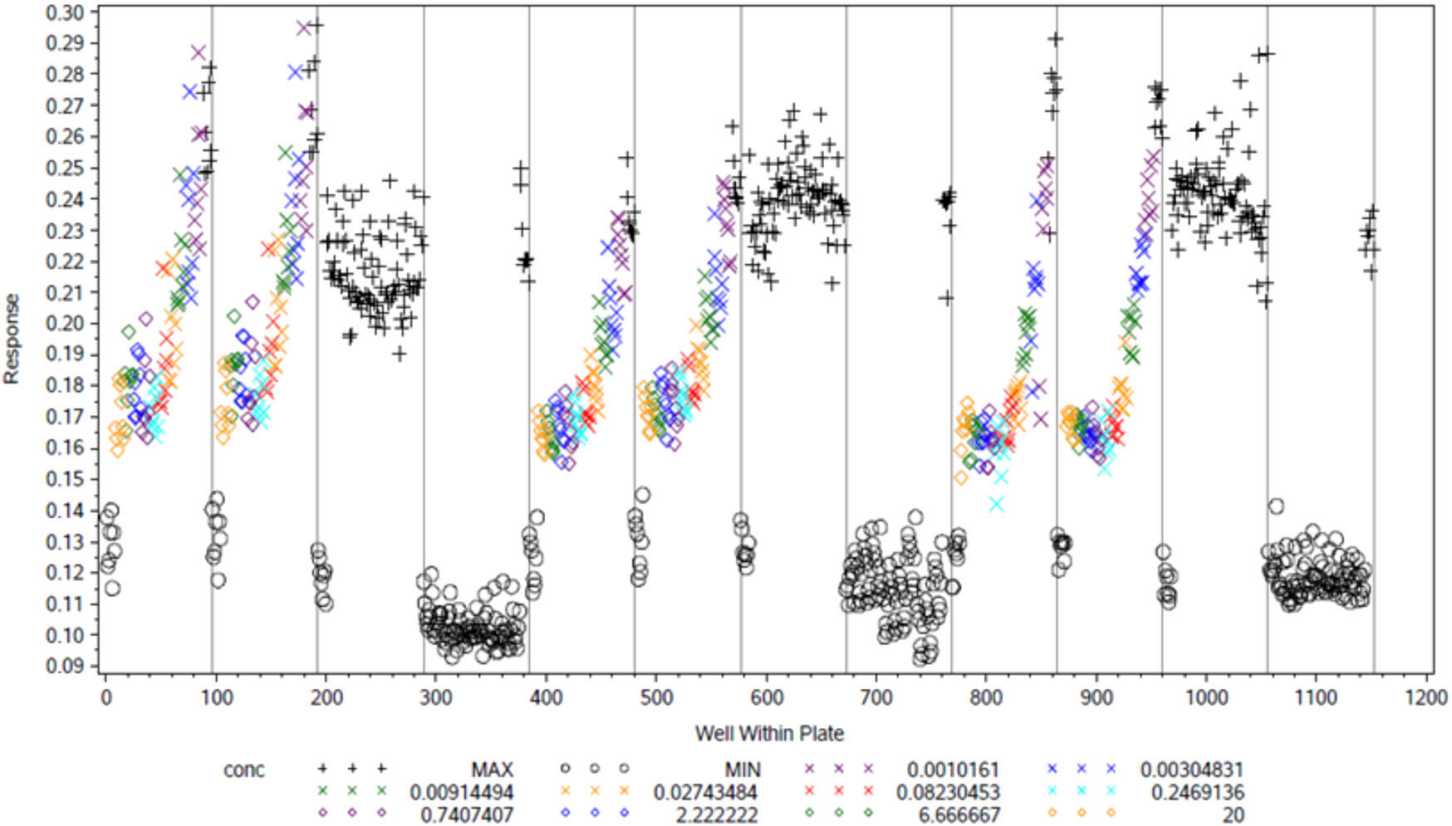
Validation of high-throughput glucose production assay. The glucose production assay of primary hepatocytes was validated in 12 96-well plates (1152 wells in total) to assess assay variability. Concentrations of insulin were titrated as indicated in the figure. The *X*-axis indicates well number and the *Y*-axis indicates raw signal value of absorbance at 570 nm

**Fig. 3:**
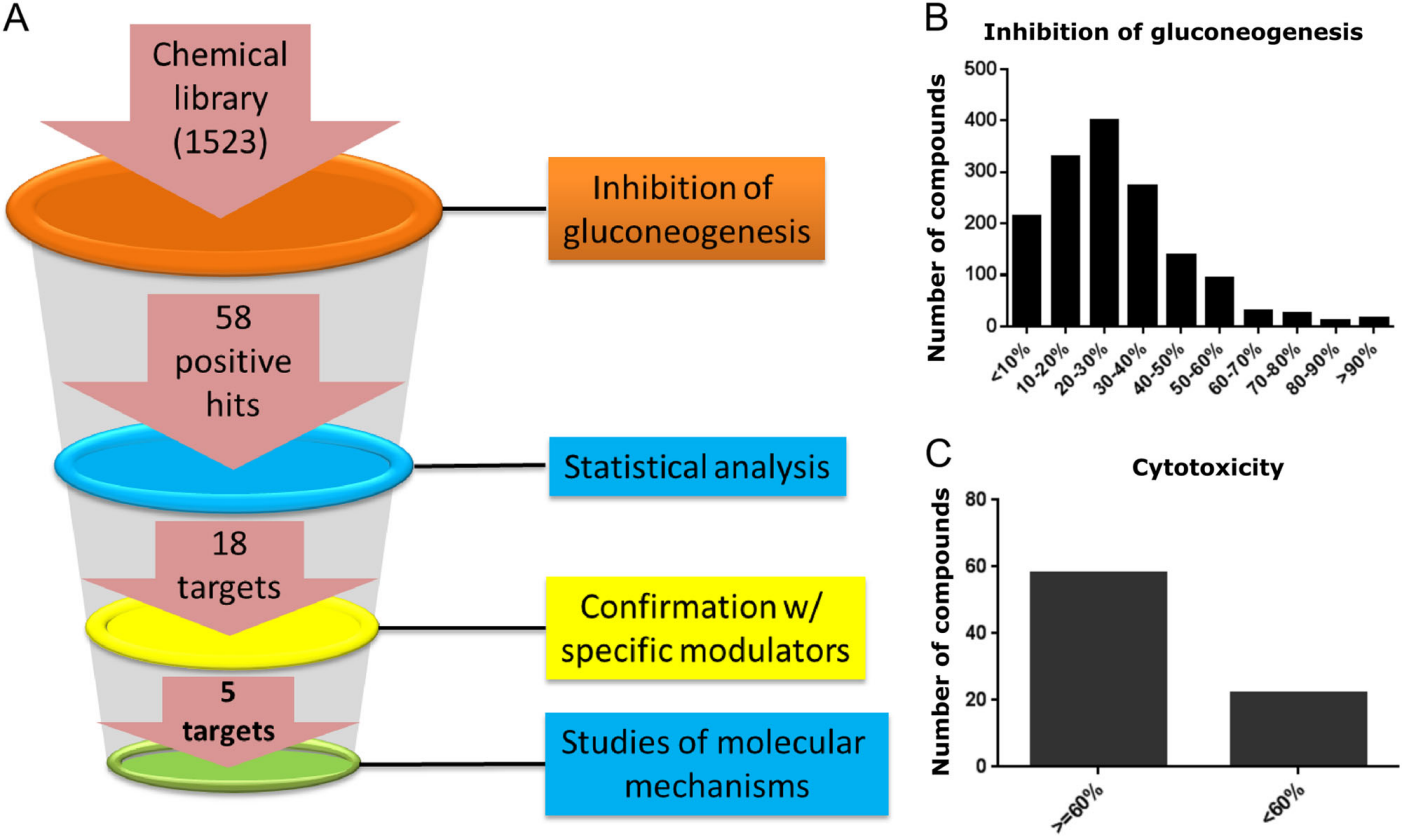
Chemical genetic approach to identification targets involved in hepatic glucose metabolism. **a** Illustration of work flow for the chemical genetic screening. **b** Overview of number of compounds with indicated inhibitory activity in the primary screen. **c** Overview of compound-caused cytotoxicity by showing cell viability test (*x*-axis) with a total of 80 compounds

We performed a validation experiment to assess the variability of the assay in high-throughput mode. Among the 12 plates tested, the range of coefficient of variation (CV) for MAX signal was between 2.4 and 7.26, the CV of MIN signal was between 2.89 and 8.95, and range of Z′ (indicator of plate uniformity) was between 0.44 and 0.78 (Fig. 2). All parameters met industrial standard for high-throughput screen. For phenotypic screen, a chemical library of 1523 small molecules was compiled and each small molecule had predefined molecular targets and selectivity profile based on prior knowledge. The effect of each compound on gluconeogenesis was tested on the human hepatocytes with or without the treatment of 1 nM insulin (Fig. 3a). An overview of compounds showing inhibitory activity (Fig. 3b) and cytotoxicity (Fig. 3c) is provided. We found that about 25% of compounds showed cytotoxicity (<60% cell viability) and these compounds were excluded from further investigation.

### Statistical analysis for target deconvolution and ranking

**Fig. 4:**
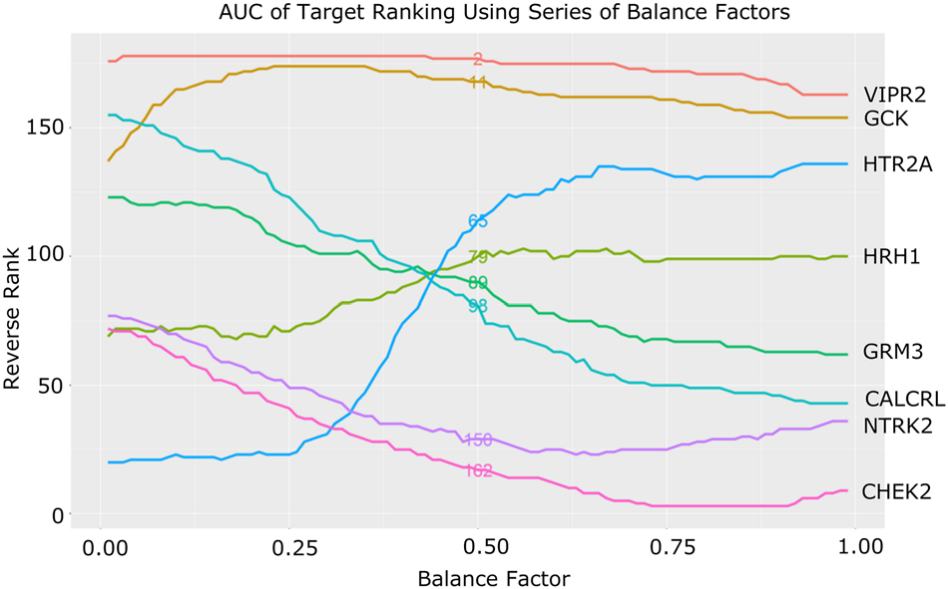
AUC of target ranking using series of balance factors. Representative targets were used to depict different scenarios resulting from balanced factors analysis. Each color line represents a target. Target names as well as their AUC rankings are shown in the figure. Neutral set of balance factor is b1 = 0.5 and b2 = 0.5, e.g., active and inactive assay both contributes half in the final ranking. Rankings of this balance factor are also displayed in the middle of each line. Overall, this neutral balance factor ranking correlates very well with the AUC ranking as we expected (Kendall’s rank correlation *τ* = 0.865)

After primary screening, 58 compounds with ≥60% inhibitory activity on glucose production and ≥60% cell viability were found to be active. In order to take both active and inactive compounds into consideration, we developed a method to statistically rank the molecular targets of the compounds in an unbiased manner (refer to “Materials and methods” section for detailed description of the method). Take HTR2A as an example to demonstrate the scenario that AUC ranking can reduce the bias of neutral balance factor ranking (Fig. 4). HTR2A ranked as 65 by neutral balance factor. As shown in the AUC plot, neutral balance factor locates near to the right plateau region of the curve. The first half of curve, e.g., b1 < 0.5, suggests this target ranked very low when contribution from active assay is low and its ranking rapidly increases with b1 in 0.25–0.5 range. In other words, its ranking is very sensitive to balance factor or less robust. Only using neutral balance factor to rank will overestimate insulin sensitization potential of HTR2A. The AUC takes the whole curve into consideration and thus ranks HTR2A as 88. Other scenarios of target deconvolution and ranking are also shown (Fig. 4).

### Identification and validation of targets involved in glucose metabolism

**Fig. 5:**
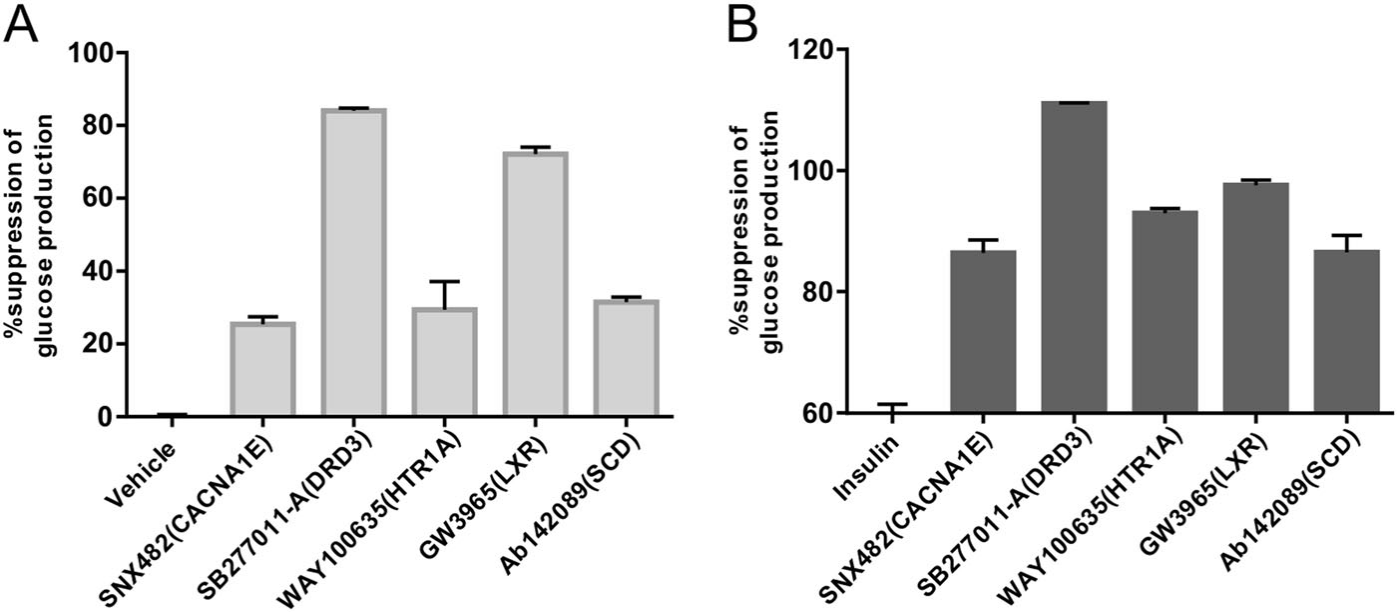
Validation of five target proteins with well-characterized specific modulators. **a** Inhibition of gluconeogenesis by indicated modulators (expressed as % of inhibition) (*n* = 3). **b** Additional inhibition of gluconeogenesis by indicated modulators on top of 1 nM insulin (*n* = 3)

After statistical analysis, top 50 targets that are potentially involved in gluconeogenesis were identified (S1 Fig). To test whether these gene targets were indeed involved in glucose homeostasis in human hepatocytes and also to test the interaction of these genes with insulin, we selected a “specific modulator” for each target protein. Based on the availability of specific modulators to us, we tested the effects of 18 modulators among the top 50 targets in gluconeogenesis with and without the presence of insulin (Table 1). The effect of CXCR2 was not confirmed due to the fact that the antagonist molecule MK7123 interfered with glucose detection method and the effects of TRPM8 and TRPV1 were not confirmed due to cytotoxicities of these molecules to primary hepatocytes. Among the rest of the 15 gene targets, we selected 5 genes (CACNA1E, DRD3, HTR1A, LXR, and SCD) to perform detailed analysis. We found that modulation of these five genes all led to suppression of gluconeogenesis (Fig. 5a). We also could confirm that modulation of all five genes clearly suppressed gluconeogenesis in the presence of insulin (Fig. 5b).

**Table 1:** Summary of 18 selected modulators

Target	Specific modulator	Type of modulation	Suppression of gluconeogenesis (%)	Suppression on top of insulin (%)
AURKB	AZD1152HQPA	Inhibitor	4	1
CACNA1E	SNX482	Blocker	25	26
CXCR2	MK7123^a^	Antagonist	NA	NA
DRD3	SB277011-A	Antagonist	84	51
ESR1	ICI182,780	Antagonist	19	7
GRM1	A841720	Antagonist	2	3
HTR1A	WAY100635	Antagonist	29	33
HTR6	SB-742457	Antagonist	15	4
HTR7	SB269970	Antagonist	12	7
LXR	GW3965	Agonist	72	38
OPRD1	SB205607	Agonist	20	4
P2RX7	A740003	Antagonist	−6	1
RARA	BMS753	Agonist	−28	−19
RPS6KB2	SL0101	Inhibitor	1	3
RXRA	CD3254	Agonist	11	3
SCD	Ab142089	Inhibitor	31	26
TRPM8	AMTB^b^	Blocker	NA	NA
TRPV1	A 784168^b^	Antagonist	NA	NA

The effects on gluconeogenesis in combination with insulin (^a^interference with detection method, ^b^cytotoxicity). The values in the table represent three replicated experiments

### Gene network analysis to understand molecular mechanisms of DRD3 and CACNA1E

**Fig. 6:**
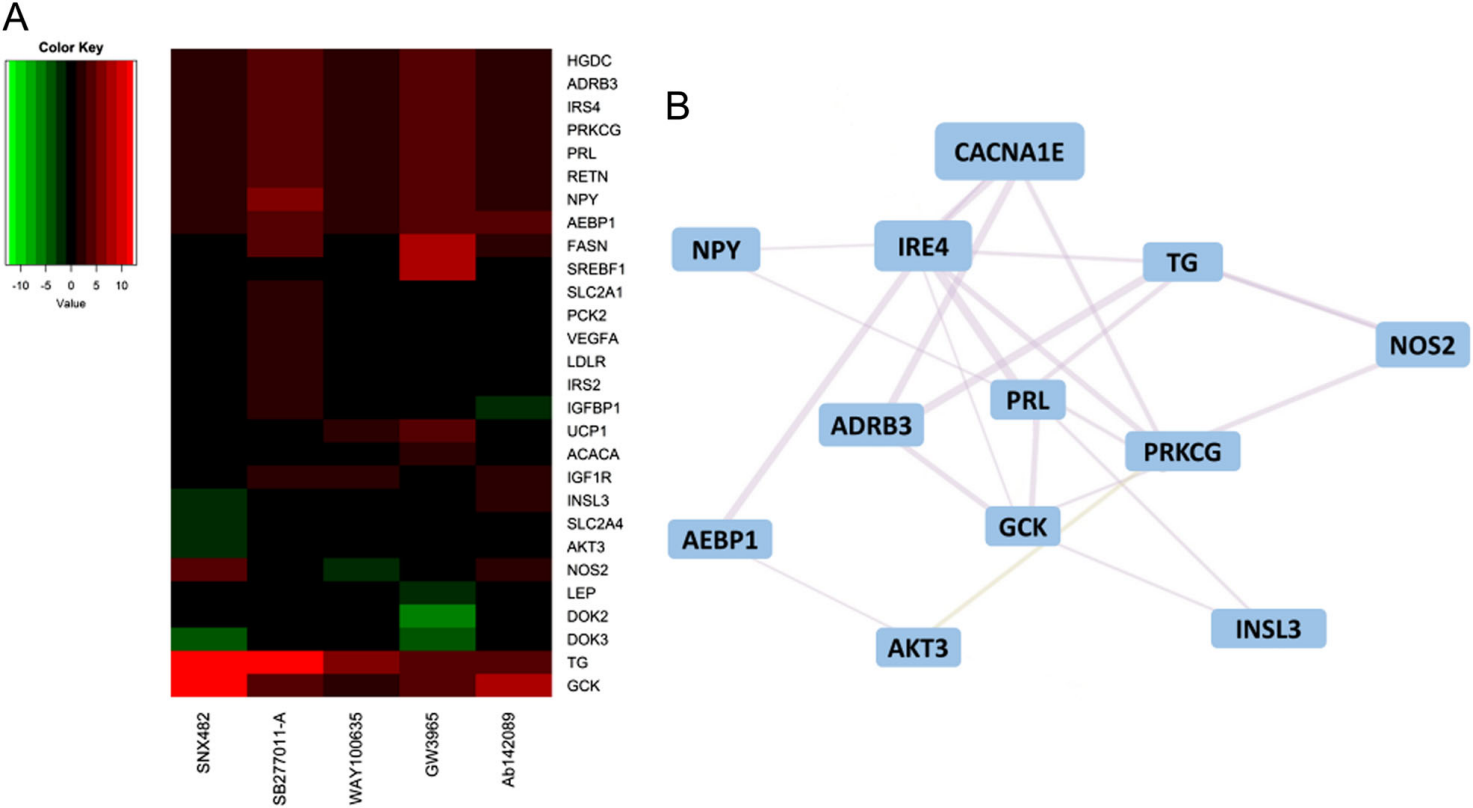
Downstream effector genes of CACNA1E and gene network analysis. **a** Genes with their expression levels differentially changed by the treatment of five compounds (SNX482, SB277011-A, WAY100635, GW3965, and Ab142089) were presented with heat map visualization using R package gplots, with color indicating fold change under treatment (cutoff at fold change more than twofold and *P* value less than 0.05). The values used to generate the heat map represent three replicas. **b** Gene interaction network built based on SNX482-targeted gene CACNA1E together with genes that were differentially expressed under SNX482 treatment

To further understand the mechanisms by which the five genes regulate gluconeogenesis in human hepatocytes, we performed a qPCR array study and monitored gene expression of a list of genes involved in glucose production and insulin sensitivity (S2 Fig and S3 Fig). Genes that were significantly (*P* < 0.000001) up or downregulated (fold change > 2) are presented in a heat map or a table (Fig. 6a and Table 2). Among these genes, we found upregulation of IRS2 and/or IRS4 and GCK (glucokinase), which are regulators of insulin sensitivity and glucose homeostasis in hepatocytes (Fig 6a and Table 2). Since the connection between CACNA1E and hepatocyte gluconeogenesis is a novel finding, we performed gene network analysis for CACNA1E to better understand which genes’ expression levels correlate with the expression of CACNA1E, and IRS4 and GCK were indeed within the co-expression gene network (Fig. 6b).

**Table 2:** Gene upregulated by DRD3 antagonist on top of insulin

Gene symbol	Fold regulation	*P* value
TG	12.31	<0.000001
PRKCG	4.45	<0.000001
IRS4	4.45	<0.000001
GCK	4.45	<0.000001
PRL	4.45	<0.000001
RETN	4.45	<0.000001
PCK2	2.42	<0.000001
IGF1R	2.35	<0.000001
IRS2	2.04	<0.000001
IGFBP1	2.02	<0.000001

Fold change (2^(−ΔΔCT)^) is the normalized gene expression (2^(−ΔCT)^) in the test sample divided by the normalized gene expression (2^(−ΔCT)^) in the control sample. Fold regulation represents fold change results in a biologically meaningful way. The *p* values are calculated based on a Student’s *t* test of the replicate 2^(−ΔCT)^ values for each gene in the control group and treatment groups. Each group contains three arrays

## Discussion

Here, we carried out a chemical genomics study aiming to identify genes involved in the regulation of gluconeogenesis in human primary hepatocytes. With a stepwise screening approach, we identified genes that are highly relevant to gluconeogenesis process in response to insulin. Limitations of this study include the limit of the chemical library, experimental design, and lack of validation in animal. Although, we used physiological levels of substrates and insulin, the in vitro cellular system cannot fully recapitulate the in vivo system. Therefore, we are not certain that the mechanism we identified will translate from human cells to human body. Nevertheless, these data suggest several mechanisms that are involved in gluconeogenesis. Both LXR and SCD have been demonstrated to be downstream effectors of insulin action and glucose metabolism in hepatocytes through regulation of lipid metabolism,^8–10^ thus identification of these targets increases our confidence about the relevance of this approach. Of note, some mechanisms that we identified have not been considered to be involved in hepatocyte glucose homeostasis. Modulation of serotonin signaling pathway has been demonstrated to be efficacious in the treatment of obesity, mostly through regulation of its functions in central nerve system.^11^ The function of serotonin in peripheral system, especially in liver, has not been extensively reported. Moore et al. showed that serotonin enhances net glucose uptake when infused into fasted dogs through portal vein.^12^ More recently, it has been reported that serotonin acts synergistically with insulin to modulate hepatic 6-phosphofructo-1-kinase and glycolysis in hepatocytes through HTR2A receptor.^13^ Our results suggest that blocking HTR1A on hepatocytes affects gluconeogenesis, possibly also in synergy with insulin. Taken together, serotonin pathway acts on glucose uptake, glycolysis, and gluconeogenesis process in hepatocytes.

DRD3 (dopamine receptor D3) is a subtype of dopamine receptor that is primarily expressed in the brain and is suggested to play a role in cognition and emotional function. Although there is a report indicating that DRD3 is associated with diabetes in human,^14^ the exact mechanism has not been elucidated so far. Here, we found that antagonism of DRD3 has a strong effect in suppressing gluconeogenesis in human hepatocytes. Further investigation is required to identify downstream signaling events that mediate its effects in hepatocytes.

Of particular interest, the function of CACNA1E in hepatocyte has not been extensively studied previously. CACNA1E is the major subunit of the voltage-dependent Ca_V_2.3 Ca^2+^ channel, which is a non-L-type high-voltage-activated calcium channel that mediates calcium entry into cells upon membrane depolarization. Ca_V_2.3 is found to be highly expressed in neuronal cells and pancreatic beta cells. So far, three studies in different ethnic groups reported association of CACNA1E variants with type 2 diabetes^15–17^ and investigation of molecular functions of the gene has been focused on pancreatic beta cells.^18,19^ Our data for the first time suggest that Ca_V_2.3 also plays a role in hepatocytes. Intracellular-free Ca^2+^ is a highly versatile second messenger that regulates a wide range of functions in every type of tissue, including liver. Many of the metabolism-related functions of the liver, including vesicular trafficking, bile secretion, glucose and lipid metabolism, and mitochondria functions, are regulated by raising intracellular Ca^2+^ in hepatocytes. Further understanding of the role of Ca_V_2.3 may provide insight into the association between calcium signaling and hepatic gluconeogenesis.

An interesting phenomenon is that among the selected 18 modulators, there is a high frequency of nuclear receptor, including LXR, RARA, and RXRA. On the other hand, the compound library is diverse to start with and is not biased toward nuclear receptors. Therefore, our finding implies the importance of nuclear receptors in the regulation of gluconeogenesis.

In conclusion, this study demonstrated a viable approach combining chemical genetics and phenotypic screening to efficiently identify molecular mechanisms in particular pathophysiology of interest. Through this approach, we identified DRD3 and CACNA1E to be potential targets for the treatment of type 2 diabetes.

## Methods

### Reagents

Human primary hepatocytes and plating media were purchased from In Vitro Technologies (Baltimore, MD; product no. M00995-P; lot no. FOS). Recombinant human insulin and glucagon, fetal bovine serum (FBS), and sodium pyruvate were from ThermoFisher Scientific (Waltham, MA). Bovine serum albumin (BSA) was from EMD Millipore (Billerica, MA). Sodium lactate, glycerol, lysine, GW3965, SB277011-A, BMS753, AZD1152HQPA, A740003, and A841720 were from Sigma-Aldrich (St. Louis, MO). SB-742457, WAY100635, Ab142089, and MK7123 were from Nanjing Norris Pharm Technology Co., Ltd (Nanjing, China). SNX482, A 784168, CD3254, SB205607, AMTB, and ICI182,780 were from R&D Systems (Minneapolis, MN). SL0101 and SB269970 were from Merck Chemicals (Shanghai, China).

### Phenotypic screen

Phenotypic screen described in the current study refers to a method for scientific experimentation performed in a high throughput and statistically meaningful manner. The readout of the method is a cellular response, represented by particular phenotype. A target refers to proteins or nucleic acids that chemical compounds bind and cause a change in its behavior or function. A compound with a desired size of effects in the phenotypic screen is defined as active. In the present investigation, the phenotype was glucose production from primary hepatocytes. The phenotypic screen in the study was composed of robotics, liquid handling devices, detectors, and data analysis process. In the present study, phenotypic screen allowed efficient test of thousands of chemical compounds. Through the process, compounds that modulate gluconeogenesis were identified. The chemical library used in the study contains a set of small molecules that inhibit or activate “druggable proteins” (proteins with potential to be modulated by a drug-like small molecule). The druggable proteins are predicted on the basis of sequence and structural similarity to the targets of existing drugs. For detailed information about druggable proteins, please refer to the knowledge portal build by Illuminating the Druggable Genome (IDG) consortium (https://druggablegenome.net/).

### Glucose production assay

Human primary hepatocytes were recovered in plating media supplemented with 10% FBS, then seeded into collagen I-coated 96-well plates (ThermoFisher Scientific, Waltham, MA) and incubated at 37 °C in a 5% CO_2_ in air atmosphere for overnight. Then, cells were starved in GOM buffer (118 mM NaCl, 4.7 mM KCl, 1.2 mM MgSO_4_, 1.2 mM KH_2_PO_4_, 25 mM NaHCO_3_, 1.2 mM CaCl_2_, pH = 7.4) plus 0.1% BSA for 6 h. After starvation, cells were further treated with different stimulants including insulin, glucagon, or compound (10 µM) ± insulin (1 nM) in the presence of gluconeogenic substrates (0.15 mM pyruvate, 1.5 mM lactate, 0.25 mM glycerol, and 2 mM lysine) for 16 h. Glucose concentration in cell culture media was determined by Amplex Red Glucose/Glucose Oxidase Assay Kit (ThermoFisher Scientific, Waltham, MA). Absorbance at 570 nm was recorded by SpectraMax® M5 Microplate Reader (Molecular Devices LLC, Sunnyvale, California). All compounds were tested at 10 µM, except SNX482 was used at 220 nM. Min control was defined by glucose production with vehicle (1% DMSO or buffer) without gluconeogenic substrates, while Max control was defined by glucose output in the presence of vehicle (1% DMSO or buffer) and gluconeogenic substrates. Final data were expressed as %Control or %Suppression of glucose production calculated by the following equations.

$$\begin{array}{ccccc}\cr & \% {\mathrm{Control}} = 100^\ast \left( {{\mathrm{Test}} - {\mathrm{Min}}} \right){\mathrm{/}}\left( {{\mathrm{Max}} - {\mathrm{Min}}} \right);{\mathrm{ }}\\\cr & \% {\mathrm{Suppression}} = 100-100^\ast \left( {{\mathrm{Test}} - {\mathrm{Min}}} \right){\mathrm{/}}\left( {{\mathrm{Max}} - {\mathrm{Min}}} \right)\cr \end{array}$$

Cell viability was determined by assessing ATP level in compound-treated cells by CellTiter Glo Luminescent Cell Viability Assay (Promega, Madison, WI).

A counter assay to assess compounds’ interference with glucose assay kit was carried out by mixing compounds with 37.5 μM glucose before proceeding glucose detection by kit. Compounds with ≥60% inhibition of gluconeogenesis were defined as “active” and compounds with <60% inhibition of gluconeogenesis were defined as “inactive”.

### Statistical analysis

We developed a two-step approach to evaluate the insulin sensitization potential of each target based on glucose production assay data. Step one was to analyze active and inactive potential separately and they were combined in step two.

As described in previous section, both active and inactive glucose production assays were performed. For a particular target, total number of compounds having been tested and number of compounds having positive results were counted. We limited the statistics analysis to 178 targets, which had at least one positive result in active assay.

In step one, data from these two types of assays were used separately to calculate the active and inactive score (*S*) for each target:

1 $$\begin{array}{ccccc}\cr & S_{i,j} = \frac{{p_{i,j}}}{{a \times w_{i,j}}},\\\cr & i = 1,2\,{\mathrm{for}}\,{\mathrm{active}}\,{\mathrm{and}}\,{\mathrm{inactive}}\,{\mathrm{assays}};j = 1, \ldots ,178\,{\mathrm{for}}\,{\mathrm{targets}}\cr \end{array}$$

where *p* was proportion of compounds that had positive results in all compounds tested in glucose production assay, *w* was the width of *p*’s Wilson intervals,^19^ *a* was adjustment factor.

In this calculation, targets with higher proportion of positive results will have higher score. For targets that have the same proportion, the ones with more evidence, e.g., more compounds being tested, will have higher score since more sample size leads to smaller confidence interval. The adjustment factor *a* was introduced to balance impacts of proportion and evidence. We found *a* = 1 worked well in this study.

Since total number of compounds tested in active and inactive assays were very different, e.g., on average a target had 35-folds more compounds tested in inactive assay than active assay, we standardized *S*_1_ and *S*_2_ separately before combining them in step two:

2 $$\begin{array}{ccccc}\cr & Z_{i,j} = \frac{{S_{i,j - }\mu _i}}{{\sigma _i}},\\\cr & i = 1,2\,{\mathrm{for}}\,{\mathrm{active}}\,{\mathrm{and}}\,{\mathrm{inactive}}\,{\mathrm{assays}};j = 1, \ldots ,178\,{\mathrm{for}}\,{\mathrm{targets}}\cr \end{array}$$

whereas *µ* and *σ* were mean and standard deviation of *S*_*i*_ calculated from all compounds for active and inactive assay.

In step two, standardized scores *Z*_*i,j*_ were combined to obtain the combined insulin sensitization score (*I*_*b,j*_). Balance factor (*b*) was applied to control contributions from active and inactive scores.

3 $$\begin{array}{l}I_{b,j} = \mathop {\sum }\limits_{i = 1,2} b_i \times Z_{i,j},\cr i = 1,2\,{\mathrm{for}}\,{\mathrm{active}}\,{\mathrm{and}}\,{\mathrm{inactive}}\,{\mathrm{assays}};j = 1, \ldots ,178\,{\mathrm{for}}\,{\mathrm{targets}}\end{array}$$

where *b*_*i*_ is the balance factor that satisfies *b*_1_ + *b*_2 _= 1.

For a particular set of balance factor *b*, for example, *b*_1_ = 0.5 and *b*_2_ = 0.5, rank of insulin sensitization score of each target was obtained.

4 $$\begin{array}{ccccc}\cr & R_{b,j} = {\mathrm{rank}}\left( {I_{b,j}} \right),\,\\\cr & b\,{\mathrm{is}}\,{\mathrm{set}}\,{\mathrm{of}}\,{\mathrm{balance}}\,{\mathrm{factor}}\,b_1 + b_2 = 1;j = 1, \ldots ,178\,{\mathrm{for}}\,{\mathrm{targets}}\cr \end{array}$$

We calculated ranks for 99 sets of balance factors (*b*_1_ = 0.01–0.99 with 0.01 increment) to cover the whole range and derived the area under curve (AUC_*j*_) for each target *j* from the *b*_1_ vs. *R*_*b,j*_ plot. Finally, insulin sensitization potential of targets were ranked by AUC_*j*_.

AUC of a few targets were shown in Fig. 4 and the full ranking table can be found in supplementary.

### Metabolic qPCR array and gene network analysis

Human primary hepatocytes in plating media with 10% FBS were seeded into collagen-coated 6-well plates (ThermoFisher Scientific, Waltham, MA) and incubated at 37 °C in a 5% CO_2_ in air atmosphere for overnight. Cells were then starved in Medium 199 (ThermoFisher Scientific, Waltham, MA) with 0.1% BSA for 4 h before treating with 1 nM insulin ± 10 µM compounds (220 nM for SNX482) for another 16 h. mRNA were collected using RNeasy plus mini kit, and then reverse transcribed by RT^2^ HT first strand kit and finally analyzed by human insulin signaling pathway PCR array according to their handbooks (Qiagen, Chatsworth, CA). Real-time PCR results were collected by ABI 7900HT instrument (Applied Biosystems, Foster City, CA). Genes with their expression levels differentially changed were presented with heat map visualization using R package. CACNA1E-associated gene changes were filled into Genemania and gene interaction network was generated with default parameters.

### Data availability statement

All relevant data are within the paper.

## Electronic supplementary material

Supplementary Figures

## Electronic supplementary material

**Supplementary information** accompanies the paper on the *npj Genomic Medicine* website (10.1038/s41525-018-0062-7).
