# Supplementary material for: Chemical genetic-based phenotypic screen reveals novel regulators of gluconeogenesis in human primary hepatocytes
Source: NPJ Genom Med. 2018 Aug 15;3:20. doi: 10.1038/s41525-018-0062-7 (PMC6093908; doi:10.1038/s41525-018-0062-7)

Supplementary Fig 1: Target ranking based on statistical analysis Shown is a detailed list of targets ranked based on the unbiased statistical model

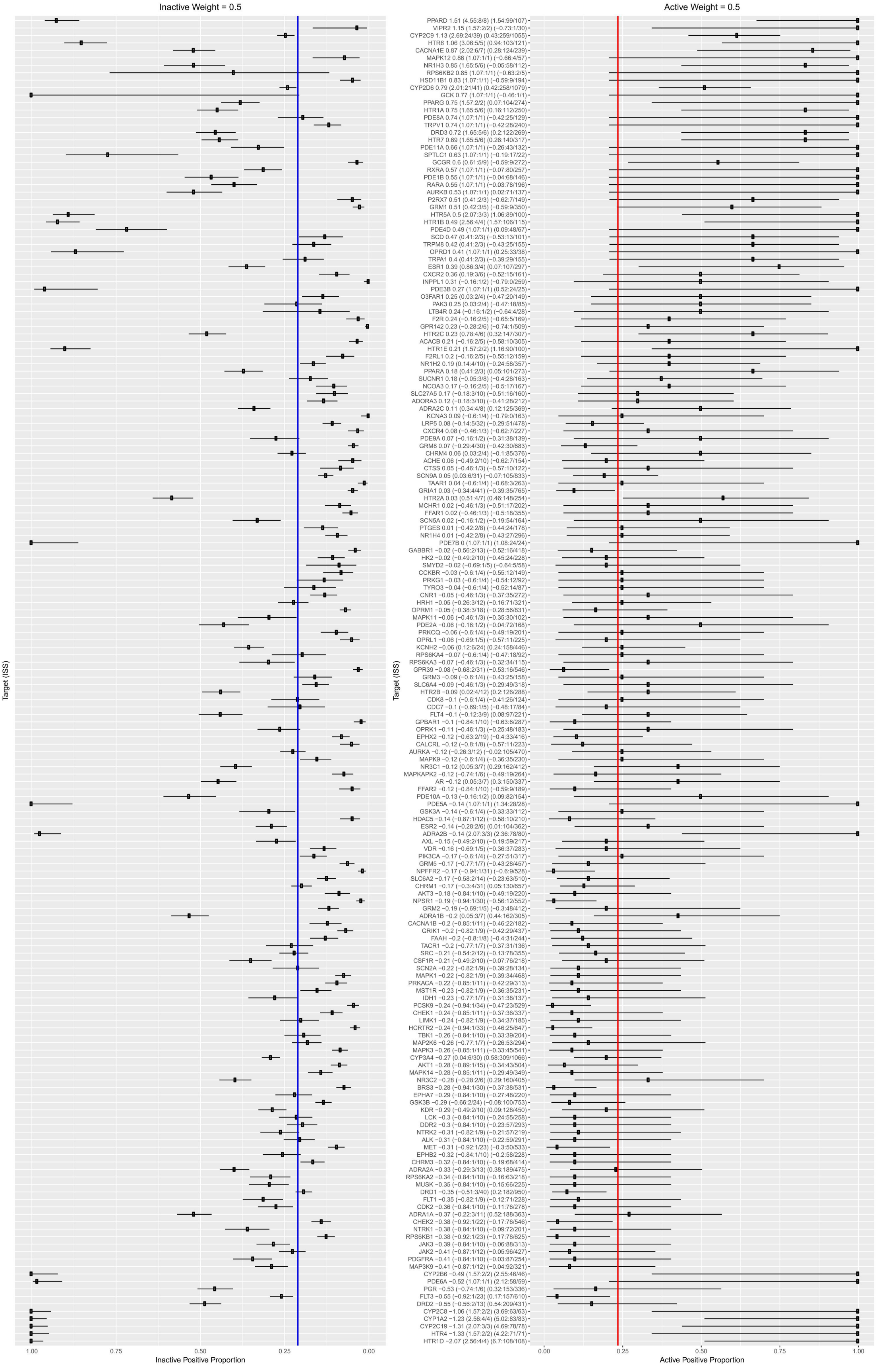

Supplementary Fig 2: Heatmap of 84 genes' expression profile of control and CACNA1E blocker-treated cells

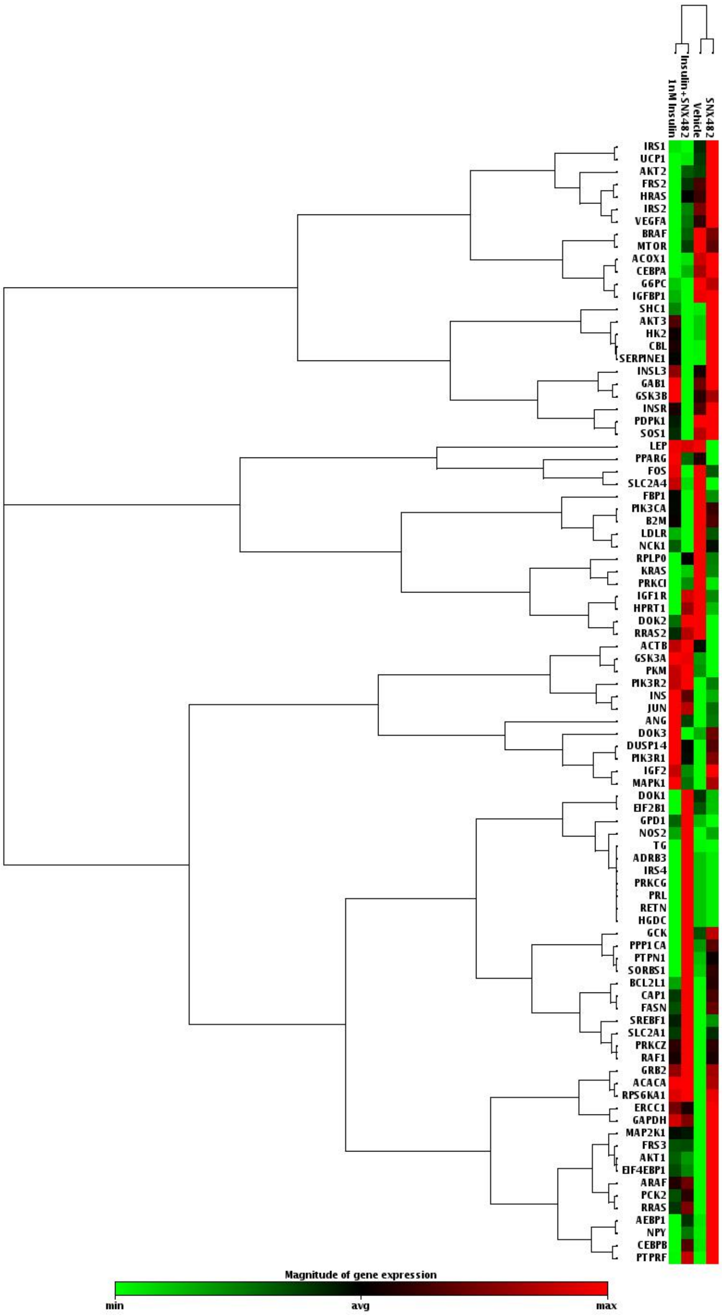

Supplementary Fig 3: Heatmap of 84 genes' expression profile of control and DRD3 antagonist-treated cells

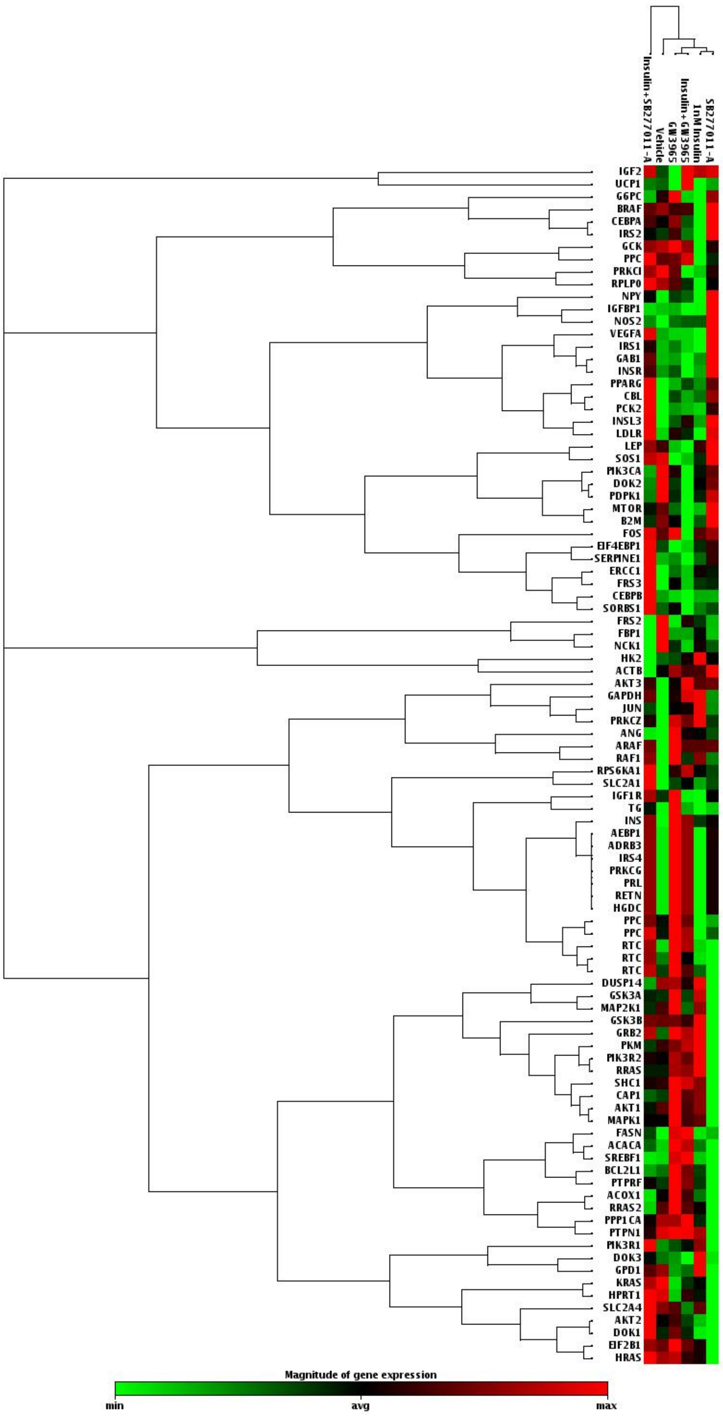

Supplement: Supplementary file 1 — Supplementary Figures [file 41525_2018_62_MOESM1_ESM.pdf]
